# Analytical solution of phosphate kinetics for hemodialysis

**DOI:** 10.1007/s00285-023-01942-4

**Published:** 2023-06-18

**Authors:** M. Andersen, K. O. Bangsgaard, J. G. Heaf, J. T. Ottesen

**Affiliations:** 1 IMFUFA, Centre for Mathematical Modeling, Human Health and Disease, Roskilde University, Roskilde, Denmark; 2 DTU Compute, Technical University of Denmark, Kongens Lyngby, Denmark; 3 Department of Nephrology, University Hospital of Zealand, Roskilde, Denmark

**Keywords:** 92-10, 92C30, 92B99, 34A30

## Abstract

Chronic kidney diseases imply an ongoing need to remove toxins, with hemodialysis as the preferred treatment modality. We derive analytical expressions for phosphate clearance during dialysis, the single pass (SP) model corresponding to a standard clinical hemodialysis and the multi pass (MP) model, where dialysate is recycled and therefore makes a smaller clinical setting possible such as a transportable dialysis suitcase. For both cases we show that the convective contribution to the dialysate is negligible for the phosphate kinetics and derive simpler expressions. The SP and MP models are calibrated to clinical data of ten patients showing consistency between the models and provide estimates of the kinetic parameters. Immediately after dialysis a rebound effect is observed. We derive a simple formula describing this effect which is valid both posterior to SP or MP dialysis. The analytical formulas provide explanations to observations of previous clinical studies.

## Introduction

Chronic kidney diseases and kidney failure cause an ongoing need of assistance for removal of toxins. For end stage renal disease, hemodialysis (HD) is the most frequent treatment modality, typically conducted thrice weekly for four hours per session at a clinic. Here the blood of the patient is circulated in an extracorporeal system where waste products and excess fluid are moved to the dialysate by the use of a semipermeable membrane. The dialysate consists of purified water, to which are added predetermined amounts of glucose, and the electrolytes sodium, potassium, calcium, magnesium, chloride and bicarbonate, but not phosphate.

**Fig. 1:**
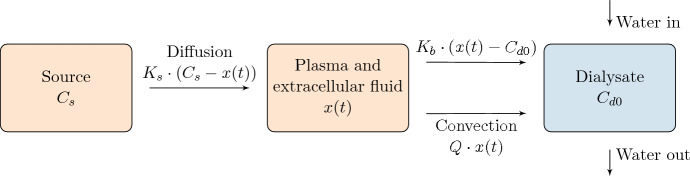
Conceptual single pass dialysis

In this standard setting, the dialysate is used only once, hence this treatment modality is coined single pass (SP) dialysis. More than 1 million people were in HD treatment in 2002—a number which has grown 7% per year (Lysaght 2002) and continues to do so (Liyanage et al. 2015). The regulation of phosphate is of particular interest, as phosphate is naturally absorbed from nutrition, and hyperphosphatemia implies an increased risk of mortality (Block et al. 1998). Even for patients receiving HD, hyperphosphatemia is prevalent (Young et al. 2004) and a major risk factor (Block et al. 1998). Therefore, it is important to understand phosphate kinetics during and between HD for patients with reduced kidney function in order to optimize treatment scheduling and potentially implement modelling of phosphate kinetics as decision support for health care professionals and patients (Heiden et al. 2013; Laursen et al. 2018). Here, we present analytical solutions to well established mathematical models of HD (describing Fig. 1 by ordinary differential equations) and estimate the kinetic parameters based on data sets of 10 HD patients.

Besides having a severe disease, the life of a HD patient implies the impractical situation of going to a dialysis unit thrice weekly. This may be resolved by having a dialysis unit at home for nocturnal use (Pierratos et al. 1998) though this requires considerable training and logistics. A transportable dialysis unit has been developed where the dialysate is being recycled in a closed system, coined multi pass (MP) dialysis (Heaf et al. 2013). This setup is easier to use than typical home dialysis, is small enough to be further transported, and reduces the amount of dialysate to less than 20% of standard treatment.

Here, we propose a mathematical model of phosphate kinetics during and between MP, solve it analytically and estimate the kinetic parameters. Clinical data of ten patients who had both been in SP and MP has previously been described clinically (Heaf et al. 2013) and is available for model calibration. We conduct parameter estimation for the two treatment modalities separately as well as combined to assess the use of MP for improved individualized estimation of the phosphate kinetics. The literature of phosphate kinetics during and between HD is not focusing on analytical solutions to the proposed models, rather numerical simulations are in focus. We present analytical solutions to some of these models for the first time including the rebound effect after HD. Though some of the calculations are straight forward they are not commonly known by nephrologists.

### Mathematical models of phosphate kinetics during HD

Mathematical models may assist in understanding phosphate kinetics in dialysis patients during or between treatment. Considering the large societal problem of malfunctioning renal function, relatively few mathematical models have been proposed since the first contribution by Sugisaki et al. (1982). Laursen et al. (2018) provide a review of mathematical models for phosphate kinetics. The model complexity ranges from one to four ordinary differential equations. As increasing model complexity comes with increasing number of parameters, the lowest order models typically provide most robust parameter estimates.

Only about 1% of the phosphate in the human body is directly accessible for HD (Pohlmeier and Vienken 2001; Agar et al. 2011) since phosphate is mainly found intracellularly. Therefore, a source term, sometimes denoted a ‘deep compartment’ is present in existing models of phosphate kinetics (Agar et al. 2011; Debowska et al. 2015; Heaf and Jensen 1994; Laursen et al. August 2015; Spalding et al. 2002; Sugisaki et al. 1983). This phosphate diffuses into the extracellular fluid at rate $K_s$. The vastness of phosphate located here justifies that this source is kept constant during dialysis time. The plasma and extracellular fluid are assumed to quickly equilibrate with the blood which goes through the dialyzer where phosphate is filtrated to the dialysate by diffusion by use of a filter with rate $K_b$ and ultrafiltration (convection) with rate *Q* (see Fig. 1). This underlines the mathematical model of SP first suggested by Agar et al. (2011), validated on a large cohorts by Agar et al. (2015), Leypoldt et al. (2013) and validated on three consecutive dialysis cycles by Debowska et al. (2015). Hence, the Agar model comprise a well established approach for dialysis modeling in agreement with clinical data. We provide an analytical solution to this model [i.e. Eq. (4) below] for any parameter values. Prior to dialysis, intra- and extracellular phosphate concentrations are assumed to be in equilibrium. The high flow of dialysate (typically 30 liters per hour) implies the phosphate concentration at the dialysate side of the filter is approximately zero. The Agar model is the simplest applied model of HD, more complex models have been suggested, which take into account a flattening or rebound of phosphate levels during HD. Though this effect may be captured (see e.g. Spalding et al. 2002), more detailed models typically have identifiability problems giving the current data. Poleszczuk et al. (2016) explain the early rebound by including a time delay in the Agar model.

## Mathematical modelling

### SP dialysis

The SP equation schematically shown in Fig. 1 is now derived. The source amount of phosphate, is so large that the source concentration of phosphate, $C_s$, can be considered constant during dialysis. Phosphate is assumed well mixed in a volume $V_b$. The concentration of phosphate in the plasma and extracelluar fluid is denoted *x*(*t*), and the volume of plasma and extracellular fluid is denoted $V_b(t)$ providing the mass of phosphate to be $m_b(t)=x(t)V_b(t)$. The mass transfer from the source compartment to the blood and plasma compartment is driven by a concentration difference given by $K_s(C_s-x(t))$, where $K_s$ is the constant phosphate transfer rate. The blood going through the dialyzer is assumed in equilibrium with extracellular fluid. The phosphate is cleared to the dialysate by diffusion across a membrane, with rate $K_b$ giving a mass loss term by $K_b(x(t)-C_{d0})$, with $C_{d0}$ being the phosphate concentration in the dialysate which is assumed to be small and constant due to the large flow of dialysate. There is a convective contribution where loss of phosphate from plasma and extracellular fluid is given by *Qx*(*t*) where the constant flow rate *Q* can be estimated by pre and post body weight of the patient divided by the treatment period, providing

1 $$\begin{aligned} {\frac{d V_b}{dt}=-Q.} \end{aligned}$$

Hence, we have for the mass balance

2 $$\begin{aligned} {\frac{d m_b}{dt}=K_s\left( C_s-x(t) \right) -K_b\left( x(t)-C_{d0} \right) -Q x(t).} \end{aligned}$$

Agar et al. (2011) omit to include the last term. Taking the shrinking volume, $V_b=V_{b0}-Qt$, into account, a differential equation of *x*(*t*) is obtained as

3 $$\begin{aligned} {\frac{d m_b}{dt}=\frac{d x(t)}{dt}V_b(t)+x(t)\frac{d V_b(t)}{dt},} \end{aligned}$$

resulting in the SP model equation

4 $$\begin{aligned} {\left( V_{b0}-Q t \right) \frac{dx(t)}{dt}=K_sC_s-\left( K_s+K_b \right) x(t)+K_b C_{d0},} \end{aligned}$$

where $V_{b0}$ is the initial volume of plasma and extracellular fluid, and $x(0)=x_0$, and the time since start of HD is denoted *t*. Typically, there is equilibrium prior to dialysis, in which case $x(0)=C_s$.

The nonautonomous SP model given by Eq. (4) can be solved by separation of variables (alternatively by a power series in in *t*)

5 $$\begin{aligned} {\int _{x_0}^x \frac{1}{K_sC_s-\left( K_s+K_b \right) X+K_b C_{d0}} dX=\int _0^t\frac{1}{V_{b0}-Q \tau }d\tau ,} \end{aligned}$$

which can be solved to give

6 $$\begin{aligned} {x(t)=\frac{K_sC_s+K_bC_{d0}}{K_s+K_b}+\left( x_0-\frac{K_sC_s+K_bC_{d0}}{K_s+K_b} \right) \left( 1-\frac{Q}{V_{b0}}t \right) ^{\frac{K_s+K_b}{Q}}.} \end{aligned}$$

For $V_{b0}\gg Qt$, the SP model may be well approximated by the simpler autonomous ordinary equation by letting $Q=0$ in Eq. (4)

7 $$\begin{aligned} {V_{b0}\frac{dx(t)}{dt}=K_sC_s-\left( K_s+K_b \right) x(t)+K_bC_{d0},} \end{aligned}$$

which has solution

8 $$\begin{aligned} x(t)=\left( x_0-\frac{K_sC_s+K_bC_{d0}}{K_s+K_b} \right) \exp \left( -\frac{K_s+K_b}{V_{b0}}t \right) +\frac{K_sC_s+K_bC_{d0}}{K_s+K_b}, \end{aligned}$$

depicting an exponential decay of phosphate approaching the steady state level $\frac{K_sC_s+K_bC_{d0}}{K_s+K_b}$ during HD. This formula solves the differential equation considered by e.g., Debowska et al. (2015) which they investigated numerically. From the well-known limit

9 $$\begin{aligned} \lim _{n\rightarrow 0}\left( 1-n x \right) ^{\frac{1}{n}}=\exp \left( -x \right) \end{aligned}$$

Equation (6) reduces to Eq. (8) for $Q\rightarrow 0$ as expected. In Fig. 3 phosphate kinetics of the SP HD predicted by Eqs. (6) and (8) are shown.

**Fig. 2:**
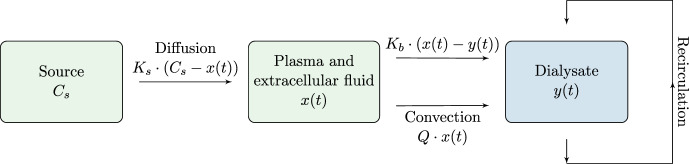
Multiple pass (MP) HD where the dialysate is recirculated

### MP dialysis

MP dialysis (Heaf et al. 2013) differs from SP dialysis by the dialysate being recycled (see Fig. 2), thus we need to take the time varying phosphate concentration in the dialysate into account, $C_d(t)=y(t)$. Hence, Eq. (2) is modified so instead of $C_{d0}$ we have *y*(*t*). The mass of phosphate in the dialysate $m_d(t)$ is given by $y(t)V_d(t)$ where $V_d$ is the volume of the dialysate compartment that grows from each initial size $V_{d0}$ be the constant rate *Q* i.e. $V_d(t)=V_{d0}+Qt$. The mass balance in the dialysate compartment is

10 $$\begin{aligned} \frac{dm_d(t)}{dt}=K_b\left( x(t)-y(t) \right) +Qx(t) \end{aligned}$$

With explicit parametrization of the dialysate volume, the relation $m_d(t)=V_d(t)y(t)$ gives

11 $$\begin{aligned} \frac{dm_d(t)}{dt}=Qy(t)+(V_{d0}+Qt)\frac{dy(t)}{dt}, \end{aligned}$$

which enables an equation for $\frac{dy(t)}{dt}$ can be obtained. The resulting MP equations are

12a $$\begin{aligned} \left( V_{b0}-Qt \right) \frac{dx(t)}{dt}&=K_sC_s-\left( K_s+K_b \right) x(t)+K_b y(t) \end{aligned}$$

12b $$\begin{aligned} \left( V_{d0}+Q t \right) \frac{dy(t)}{dt}&=\left( K_b+Q \right) \big (x(t)-y(t)\big ), \end{aligned}$$

with initial conditions $x(0)=x_0$, $y(0)=y_0$.

In the HD equipment, the dialysate and blood stream run in long thin channels separated by a filter. The inlet dialysate concentration for SP is 0 throughout the treatment whereas it increases for MP. The outlet concentration equals the inlet concentration plus the accumulated amount per volume filtered from the blood by diffusion across the membrane. The contribution from the blood decreases during dialysis since the concentration in the blood decreases (and for MP the inlet concentration increases). We assume a linear decrease in the blood phosphate concentration and a corresponding linear increase in the dialysate concentration along the channels. Thus, the spatial average concentration for the dialysate in the channels at a given time becomes the outlet concentration plus the inlet concentration divided by 2. This deviates for the conventional approach where solely the inlet concentration is used. However, the conventional approach is a good approximation to our more accurate approach for small concentrations as seen in up to 8 h of HD sessions. For completeness, we have tested the different scenarios for $C_{d0}$ and found that the choice has insignificant impact on the simulations and the estimates.

### Analytical solution to the MP model

To solve the MP model in Eq. (12), we first make the ansatz that *x*(*t*) and *y*(*t*) can be expressed as power series and derive a formula for the coefficients in the power series. Then, we investigate the radius of convergence for the power series showing that the power series is meaningful.

Ansatz

13a $$\begin{aligned} x(t)&=\sum _{k=0}^\infty \alpha _kt^k \end{aligned}$$

13b $$\begin{aligned} y(t)&=\sum _{k=0}^\infty \beta _kt^k. \end{aligned}$$

The initial conditions imply

14a $$\begin{aligned} \alpha _0&=x_0 \end{aligned}$$

14b $$\begin{aligned} \beta _0&=y_0. \end{aligned}$$

The task is then to find expressions for the remaining unknown coefficients $\alpha _i$ and $\beta _i$ and to prove the resulting series converge. Within the radius of convergence, the series can be differentiated term by term resulting in

15a $$\begin{aligned} \frac{dx(t)}{dt}&=\sum _{k=1}^\infty k\alpha _kt^{k-1}=\sum _{k=0}^\infty \left( k+1 \right) \alpha _{k+1}t^{k} \end{aligned}$$

15b $$\begin{aligned} \frac{dy(t)}{dt}&=\sum _{k=1}^\infty k\beta _kt^{k-1}=\sum _{k=0}^\infty \left( k+1 \right) \beta _{k+1}t^{k}. \end{aligned}$$

Inserting Eqs. (13) and (15) in Eq. (12) we get

16a $$\begin{aligned} V_{b0}\sum _{k=0}^\infty \left( k+1 \right) \alpha _{k+1}t^k-Q\sum _{k=1}^\infty k\alpha _kt^k&=K_sC_s-\left( K_s+K_b \right) \sum _{k=0}^\infty \alpha _kt^k+K_b\sum _{k=0}^\infty \beta _kt^k \end{aligned}$$

16b $$\begin{aligned} V_{d0}\sum _{k=0}^\infty \left( k+1 \right) \beta _{k+1}t^k+Q\sum _{k=1}^\infty k\beta _kt^k&=\left( K_b+Q \right) \sum _{k=0}^\infty \left( \alpha _k-\beta _k \right) t^k. \end{aligned}$$

Since the coefficients of the zero polynomial are all zero, we obtain recursion formalas for the hitherto unknown coefficients in Eq. (13),

17a $$\begin{aligned} \alpha _1&=\frac{K_sC_s-\left( K_s+K_b \right) \alpha _0+K_b\beta _0}{V_{b0}} \end{aligned}$$

17b $$\begin{aligned} \alpha _{k+1}&=\frac{Qk\alpha _k-\left( K_s+K_b \right) \alpha _k+K_b\beta _k}{V_{b0}\left( k+1 \right) },\quad \text {for}\quad k\ge 1 \end{aligned}$$

17c $$\begin{aligned} \beta _{k+1}&=\frac{-Qk\beta _k+\left( K_b+Q \right) \left( \alpha _k-\beta _k \right) }{V_{d0}\left( k+1 \right) },\quad \text {for}\quad k\ge 0, \end{aligned}$$

with $\alpha _0$ and $\beta _0$ given by Eq. (14). The Eqs. (17b), (17c) can conveniently be put in matrix form

18 $$\begin{aligned} {\varvec{\gamma }}_{k+1} = B_k{\varvec{\gamma }}_k, \quad \text {for}\quad k\ge 1 \end{aligned}$$

with

19 $$\begin{aligned} B_k=\frac{1}{k+1}\begin{bmatrix} \frac{Qk-\left( K_s+K_b \right) }{V_{b0}}&{}\frac{K_b}{V_{b0}}\\ \frac{K_b+Q}{V_{d0}}&{}-\frac{Qk+K_b+Q}{V_{d0}} \end{bmatrix}, \end{aligned}$$

and

20 $$\begin{aligned} {{\varvec{\gamma }}_k=\begin{bmatrix} \alpha _{k}\\ \beta _{k} . \end{bmatrix}} \end{aligned}$$

Repeated use of Eq. (18) gives the expression for the *k*’th coefficients,

21 $$\begin{aligned} {\varvec{\gamma }}_{k}=B_{k-1}B_{k-2}\ldots B_1{\varvec{\gamma }}_1. \end{aligned}$$

Left is to identify the radius of convergence of the power series using Weierstrass’ *M*-test. Clearly $B_k$ has a limit for $k\rightarrow \infty$,

22 $$\begin{aligned} B=\lim _{k\rightarrow \infty } B_k=\begin{bmatrix} \frac{Q}{V_{b0}}&{}0\\ 0&{}-\frac{Q}{V_{d0}} \end{bmatrix}, \end{aligned}$$

with eigenvalues $\frac{Q}{V_{b0}}$ and $-\frac{Q}{V_{d0}}$. For any $\epsilon >0$ it is hence possible to find $N>0$ s.t for all $k\ge N$ the eigenvalues of $B_k$ are in the set *S*

23 $$\begin{aligned} S=\begin{bmatrix} \frac{Q}{V_{b0}}-\epsilon ,\frac{Q}{V_{b0}}+\epsilon \end{bmatrix}\times \begin{bmatrix} -\frac{Q}{V_{d0}}-\epsilon ,-\frac{Q}{V_{d0}}+\epsilon \end{bmatrix}. \end{aligned}$$

This provides a uniform bound on the absolute value of the eigenvalues of $B_k$ for any $k\ge N$, ${\tilde{\lambda }}=\max \{\frac{Q}{V_{b0}}+\epsilon ,\frac{Q}{V_{d0}}+\epsilon \}$. A power series converges if the tail converges and the above limit provides information about this. By repeated use of

24 $$\begin{aligned} ||B_k {\varvec{\gamma }}_k||\le {\tilde{\lambda }} ||{\varvec{\gamma }}_k||,\quad \text {for}\quad k\ge N, \end{aligned}$$

we obtain

25 $$\begin{aligned} ||{\varvec{\gamma }}_{N+l}||\le {\tilde{\lambda }}^{l-1} ||{\varvec{\gamma }}_N||,\quad \text {for}\quad l\ge 1. \end{aligned}$$

From definition (20)

26 $$\begin{aligned} |\alpha _k|\le ||{\varvec{\gamma }}_{k}||, \end{aligned}$$

the tail of the series for *x*(*t*) can be estimated

27 $$\begin{aligned} |\sum _{k=N+1}^\infty \alpha _kt^k|\le \sum _{l=1}^\infty |\alpha _{N+l}||t^{N+l}| \le ||{\varvec{\gamma }}_{N}||{\tilde{\lambda }}^{-1}|t^N| \sum _{l=1}^\infty |{\tilde{\lambda }}t|^l. \end{aligned}$$

The series on the right-hand side is recognized as the geometric series which is convergent if and only if $|{\tilde{\lambda }}t|<1$. Hence by Weierstrass M-test $\sum _{k=0}^\infty \alpha _kt^k$ converges uniformly for $t\in [0,\frac{1}{{\tilde{\lambda }}})$. Similar considerations apply to the series for *y*(*t*).

In summary, a sufficient criterion for the ansatz (13) with coefficients giving by (17) to hold is $0<t<\min \{\frac{V_{b0}}{Q},\frac{V_{d0}}{Q} \}$. As $t=\frac{V_{b0}}{Q}$ corresponds to a complete depletion of the plasma and extracellular fluid compartment this is a reasonable requirement. A benefit of the explicit solution is hence to show a large interval of existence in time and explicit parameter dependence on the solution. Furthermore, numerical integration algorithms do not provide an absolute error for the numerical approximation, but this is obtained from Taylor’s remainder formula for a given truncation of the analytical series solution. Hence, with the truncated, explicit solution the maximal error to the real solution is estimated, which cannot be obtained from direct numerical approximations.

The total dialysis time, *T*, is typically four hours for SP and eight hours for MP. This can be used to give an upper bound on the error of truncation of the exact, analytical solutions giving by a power series. An upper bound on the error when keeping only *N* terms of the series is given by Lagrange’s Remainder Theorem.

28a $$\begin{aligned} {\textit{error}}_{x,N}(t)&\le |\alpha _{N+1}|t^{N+1} \end{aligned}$$

28b $$\begin{aligned} {\textit{error}}_{y,N}(t)&\le |\beta _{N+1}|t^{N+1}. \end{aligned}$$

**Fig. 3:**
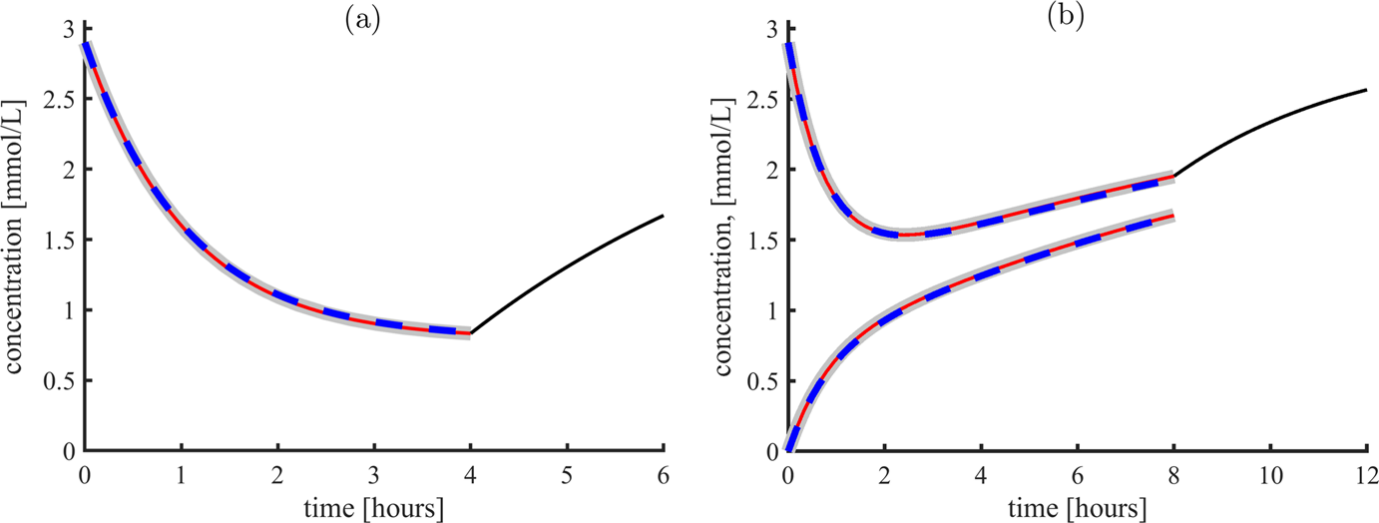
Analytical solutions to the SP and MP models of HD with two hours post dialysis rebound. **a** Four hours SP dialysis, grey curve is numerical integration of Eq. (4), red curve is the formula (6) and blue is the formula when neglecting ultrafiltration, Eq. (8). Black curve is the rebound after dialysis, given by Eq. (44). **b** Eight hours MP analysis. Curves starting at (0, 2.9) are phosphate concentration in serum, curves starting at (0, 0) are phosphate in dialysate. Grey curves correspond to direct numerical integration of the differential equations. Red curve is the analytical series solution including the first 27 terms. Blue curve is the analytical solution with $Q=0$, Eq. (42). Black curve is the rebound after treatment, Eq. (44). Parameter values in both panels are $K_s=2.6$ L/h, $C_s=2.9$ mmol/L, $K_b=6.9$ L/h, $Q=0.23$ L/h, $V_{b0}=10$ L, $x_{0}=C_{s}, y_{0}=0$ (color figure online)

Hence, one can compute how many terms in the series that must be included to guarantee a prespecified accuracy. For example, a truncation error of at most 0.01 mmol/L is guaranteed by including 27 terms for the specified rate parameters, see Fig. 3.

### Explicit solution of MP model with $Q=0$

Assuming *Q* is negligible, Eq. (12) reduces to

29a $$\begin{aligned} V_{b0}\frac{dx(t)}{dt}&=K_sC_s-\left( K_s+K_b \right) x(t)+K_b y(t) \end{aligned}$$

29b $$\begin{aligned} V_{d0}\frac{dy(t)}{dt}&=K_b\left( x(t)-y(t) \right) . \end{aligned}$$

In matrix notation with ${\varvec{x}}=(x,y)^T$, ${\varvec{b}}=\left( \frac{K_sC_s}{V_{b0}},0 \right) ^T$ and

30a $$\begin{aligned} A= & {} \begin{bmatrix} -\frac{K_s+K_b}{V_{b0}} &{} \frac{K_b}{V_{b0}} \\ \frac{K_b}{V_{d0}} &{} -\frac{K_b}{V_{d0}} \end{bmatrix}, \end{aligned}$$

Equation (29) can be written

31 $$\begin{aligned} \frac{d{\varvec{x}}}{dt}=A{\varvec{x}}+{\varvec{b}}. \end{aligned}$$

Both eigenvalues of *A* are real and negative since *A* has negative trace and positive determinant, and thus the matrix *A* is invertible with eigenvalues given by

32a $$\begin{aligned} \lambda _1&=\frac{1}{2}\left( -\frac{K_s+K_b}{V_{b0}}-\frac{K_b}{V_{d0}}+\sqrt{\left( -\frac{K_s+K_b}{V_{b0}}+\frac{K_b}{V_{d0}} \right) ^2+4\frac{K_b^2}{V_{b0}V_{d0}}} \right) \end{aligned}$$

32b $$\begin{aligned} \lambda _2&=\frac{1}{2}\left( -\frac{K_s+K_b}{V_{b0}}-\frac{K_b}{V_{d0}}-\sqrt{\left( -\frac{K_s+K_b}{V_{b0}}+\frac{K_b}{V_{d0}} \right) ^2+4\frac{K_b^2}{V_{b0}V_{d0}}} \right) . \end{aligned}$$

Introducing

33 $$\begin{aligned} {\varvec{\omega }}={\varvec{x}}+A^{-1}{\varvec{b}} \end{aligned}$$

with

34 $$\begin{aligned} A^{-1}{\varvec{b}}=-C_s \begin{bmatrix} 1 \\ 1 \end{bmatrix}, \end{aligned}$$

Equation (31) can equivalently be written

35 $$\begin{aligned} \frac{d{\varvec{w}}}{dt}=A{\varvec{w}}. \end{aligned}$$

This may be solved by linear algebra, but for completeness we will use the recursion formula which provides a series that can be explicitly evaluated in closed form in this case.

Let

36a $$\begin{aligned} \omega _1(t)&=\sum _{k=0}^\infty {\hat{\alpha }}_kt^k \end{aligned}$$

36b $$\begin{aligned} \omega _2(t)&=\sum _{k=0}^\infty {\hat{\beta }}_kt^k, \end{aligned}$$

and ${\varvec{{\hat{\gamma }}}}_k=({\hat{\alpha }}_k, {\hat{\beta }}_k)$, then from the recurrence formulas we obtain

37 $$\begin{aligned} { {\varvec{{\hat{\gamma }}}}_{k+1} = \frac{1}{k+1}A{\varvec{{\hat{\gamma }}}}_k, \quad \text {for}\quad k\ge 0,} \end{aligned}$$

which can be iteratively applied to obtain

38 $$\begin{aligned} { {\varvec{{\hat{\gamma }}}}_{k} = \frac{1}{k!}A^k{\varvec{{\hat{\gamma }}}}_0, \quad \text {for}\quad k\ge 0.} \end{aligned}$$

The feasibility of an analytical solution is due to *A* being a constant matrix i.e., not dependent on *k*, which means that standard linear algebra techniques can be applied. Let *D* be a diagonal 2*x*2 matrix with $\lambda _1$ and $\lambda _2$ at the diagonal entries and *P* be a matrix with the corresponding eigenvectors as the columns. Then, $A=PDP^{-1}$ which applied to Eq. (38) gives

39 $$\begin{aligned} { t^k{\varvec{{\hat{\gamma }}}}_{k} = P\begin{bmatrix} \frac{\left( \lambda _1 t \right) ^k}{k!}&{}0\\ 0&{}\frac{\left( \lambda _2 t \right) ^k}{k!} \end{bmatrix}P^{-1}{\varvec{{\hat{\gamma }}}}_0, \quad \text {for}\quad k\ge 0.} \end{aligned}$$

The summation for all *k* [Eq. (36)] can be explicitly obtained from the well-known series of the exponential function

40 $$\begin{aligned} {\begin{bmatrix} w_1(t)\\ w_2(t) \end{bmatrix}= P\begin{bmatrix} \exp (\lambda _1t)&{}0\\ 0&{}\exp (\lambda _2t) \end{bmatrix}P^{-1}{\varvec{{\hat{\gamma }}}}_0. }\end{aligned}$$

Transforming back to original coordinates by use of Eq. (33) we obtain

41 $$\begin{aligned} {\begin{bmatrix} x(t)\\ y(t) \end{bmatrix}= P\begin{bmatrix} \exp (\lambda _1t)&{}0\\ 0&{}\exp (\lambda _2t) \end{bmatrix}P^{-1} \begin{bmatrix} x_0-C_s \\ y_0-C_s \end{bmatrix}+C_s \begin{bmatrix} 1 \\ 1 \end{bmatrix}. } \end{aligned}$$

Thus, for $Q=0$, the phosphate concentration in serum, as well as in the dialysate, is well represented by the sum of two exponential functions plus a constant term.

Equation (41) can be written explicitly by

42a $$\begin{aligned} x(t)&=\frac{V_{d0}\lambda _1+K_b}{K_bV_{d0}\left( \lambda _1-\lambda _2 \right) }\Big ( K_b\left( x_0-C_s \right) -\left( V_{d0}\lambda _2+K_b \right) \left( y_0-C_s \right) \Big )\exp \left( \lambda _1 t \right) \nonumber \\&\quad +\frac{V_{d0}\lambda _2+K_b}{V_{d0}\left( \lambda _1-\lambda _2 \right) }\Big ( -K_b\left( x_0-C_s \right) +\left( V_{d0}\lambda _1+K_b \right) \left( y_0-C_s \right) \Big )\exp \left( \lambda _2 t \right) +C_s \end{aligned}$$

42b $$\begin{aligned} y(t)&=\frac{1}{V_{d0}\left( \lambda _1-\lambda _2 \right) }\Big ( K_b\left( x_0-C_s \right) -\left( V_{d0}\lambda _2+K_b \right) \left( y_0-C_s \right) \Big )\exp \left( \lambda _1 t \right) \nonumber \\&\quad +\frac{1}{V_{d0}\left( \lambda _1-\lambda _2 \right) }\Big ( -K_b\left( x_0-C_s \right) +\left( V_{d0}\lambda _1+K_b \right) \left( y_0-C_s \right) \Big )\exp \left( \lambda _2 t \right) +C_s. \end{aligned}$$

## The rebound effect post treatment

The total dialysis time, *T*, is typically four hours or eight hours. After the dialysis treatment, the phosphate kinetics is governed by diffusion between the source compartment and the serum fluid leading to the differential equation

43 $$\begin{aligned} \text {For}\;t\ge T: \quad V_{b0}\frac{dx(t)}{dt}=K_sC_s-K_sx(t),\quad \text {with } x(T)=x_T, \end{aligned}$$

which is easily solved

44 $$\begin{aligned} x(t)= \left( x_T-C_s \right) \exp \left( -\frac{K_s}{V_{b0}}\left( t-T \right) \right) +C_s,\quad \text {for}\quad t>T. \end{aligned}$$

Prior to dialysis, the phosphate concentration in the source and in the serum is expected to balance, and during dialysis the serum phosphate is reduced, hence $x_T-C_s<0$ so the formula Eq. (44) depicts growth. All analytical solutions of the SP and MP model, with and without ultrafiltration is shown in Fig. 3, including the rebound curve after dialysis Eq. (44). From this, ultrafiltration is negligible and henceforth, we will assume $Q=0$.

## Parameter estimation

From Heaf et al. (2013) we have data of 10 HD patients receiving SP treatment at one session and MP treatment at another session, which we will use to estimate the parameters of the SP and MP model. Based on the previous analysis we set $Q = 0$, and repeat the SP [Eq. (7)] and MP [Eq. (29)],

45 $$\begin{aligned} V_{b0} \frac{dz(t)}{dt}&= C_s K_s-(K_s+K_b) z(t)+K_b C_{d0} \end{aligned}$$

46 $$\begin{aligned} V_{b0} \frac{dx(t)}{dt}&= C_s K_s-(K_s+K_b) x(t)+K_b y(t) \end{aligned}$$

47 $$\begin{aligned} V_{d0}\frac{dy(t)}{dt}&= K_b (x(t)- y(t)). \end{aligned}$$

We give the concentration of phosphate in the blood the new label, *z*(*t*), for the SP model, with initial condition, $z_0=z(0)$, to be able to estimate the parameters of the model by solely SP data, solely MP data, or both in combination which is denoted coupled pass (CP).

**Table 1:** Individual estimates of the constant concentration of phosphate in the dialysate for SP, $C_{d0}$, the dialysate volume for MP, $V_{d0}$, and the initial estimate for $V_{b0}$ based on somewhat inaccurate clinical measure of extracellular volume

	Patient									
	1	2	3	4	5	6	7	8	9	10
$C_{d0}$ (mmol/L)	0.16	0.12	0.11	0.16	0.18	0.21	0.14	0.09	0.14	0.09
$V_{d0}$ (L)	22.61	23.00	26.57	31.93	28.51	20.15	23.42	14.24	28.59	23.25
$V_{b0}$ (L)	16.95	17.78	17.25	21.20	18.32	15.00	15.42	13.50	20.21	18.16

The phosphate concentration in the blood and dialysate have been measured at baseline and every hour during HD. In addition, the volume of dialysate, $V_{d0}$, was measured. The individual estimates of $V_{d0}$ for MP, and the constant dialysate concentration, $C_{d0}$, for SP are listed in Table 1 for each patient since these can be estimated independently of and previous to the remaining parameters. The parameters of the SP, MP and CP model are structurally identifiable conditioned to the parameters in this table.

We consider the initial conditions $z_0$ and $x_0$ to be parameters of the model since they are prone to measurement noise, just as the measurements at time $t >0$. Initially, we performed estimation with $y_0$ as a parameter of the model, i.e., we allowed $y_0$ to deviate from zero. The deviation from zero has the interpretation that we consider $y_0$ as a spatial average of concentration in the dialysate from inlet to outlet of the filter. However, allowing $y_0$ to deviate from zero, did not have significant impact on the estimates nor the fits of the model to data. Thus, we ended up by fixating $y_0 = 0$ to reduce the complexity of the model.

The concentration of phosphate in the blood will be close to steady state, $C_s$, some time after ended treatment. Hence, we reduce the number of parameters by assuming that the patient is at steady state when dialysis is initiated, i.e. $C_s=z_0$ and $C_s= x_0$ for SP and MP, respectively. For CP, we have two measurements of the concentration in the blood at time $t = 0$. Here we make the model assumption that $C_s$ is equal to the largest measurement at time $t =0$, i.e.,

$$\begin{aligned} C_s = \left\{ \begin{array}{ll} z_0 &{}\quad \text {if}\; z_0 \ge x_0\\ x_0 &{}\quad \text {otherwise} \end{array} \right. . \end{aligned}$$

The system of differential Eqs. (45–47) is non-linear in the parameters after division with the volumes $V_{b0}$ and $V_{d0}$ whenever these are estimated. Keeping these fixed in the first step render the differential equations linear and a uniquely determined pre-estimate for the remaining parameters are obtained. Thus, these pre-estimated parameters are used as initial guesses along with the clinically less accurate measurement for the volumes in a subsequent non-linear parameter estimation procedure. In the following we elaborate on this approach. To estimate the parameters, we use the non-linear least squares estimate (Dattner and Gugushvili 2018; Qiu et al. 2015). The objective function for the parameter estimation of the unknown parameter vector $\eta$ is,

48 $$\begin{aligned} \text {RMSE}(\eta ) = \sqrt{\frac{1}{dn}\sum _{j=1}^d\sum _{i=1}^n \left( Y_{ij}-{\hat{X}}_j(t_i,\eta )\right) ^2}, \end{aligned}$$

where $d = {1,2,3}$ depending on whether we consider SP, MP or CP, respectively, $Y_{ij}$ are data points and ${\hat{X}}(t)$ denote the numerical model solution, e.g., obtained by Runge Kutta 45. We consider the initial conditions to be parameters of the system since they are likewise prone to be affected by measurement noise.

The solution ${\hat{X}}(t)$ is non-linear in the parameters $\eta$, therefore we need an iterative procedure to compute the non-linear least square estimate. We use MatLab’s fmincon for the non-linear optimization process. Moreover, we add upper and lower bounds to our non-linear optimization procedure to ensure that the computed parameters are within a physiological meaningful range. The enforced upper and lower bounds are listed in Table 2. Estimates for the uncertainties on the estimated parameters are obtained using the Hessian matrix *H* of the cost function evaluated at the optimum. More precise, as uncertainties we take the 95% confidence interval for the estimates $\theta$, calculated from the square root of the diagonal elements of the covariance matrix (Aster et al. 2019). The obtained parameter values and uncertainties appear in Tables 3, 4 and 5 for all estimated parameters and all patients.

**Table 2:** Lower and upper bounds for the non-linear parameter estimation

	$K_s$ (L/h)	$K_b$ (L/h)	$V_{b0}$ (L)	$z_0$ (mmol/L)	$x_0$ (mmol/L)
Lower bound	0	0	0	0	0
Upper bound	30	30	60	5	5

**Table 3:** SP parameter values corresponding to Fig. 4 with 95% confidence interval for each parameter estimate specified

Patient	$C_s$ (mmol/L)	$K_s$ (L/h)	$K_b$ (L/h)	RMSE
1	$1.98 \pm 0.01$	$8.51 \pm 0.22$	$15.47 \pm 0.28$	0.01
2	$1.26 \pm 0.01$	$13.75 \pm 1.23$	$17.4 \pm 1.28$	0.02
3	$1.18 \pm 0.01$	$21.16 \pm 1.54$	$15.01 \pm 0.98$	0.01
4	$1.97 \pm 0.00$	$10.88 \pm 0.24$	$12.37 \pm 0.19$	0.01
5	$1.75 \pm 0.08$	$14.10 \pm 5.26$	$16.47 \pm 5.03$	0.05
6	$2.75 \pm 0.01$	$4.74 \pm 0.09$	$10.75 \pm 0.11$	0.01
7	$1.62 \pm 0.14$	$6.18 \pm 8.75$	$2.77 \pm 2.55$	0.08
8	$1.19 \pm 0.02$	$8.73 \pm 1.15$	$11.50 \pm 1.18$	0.02
9	$1.22 \pm 0.01$	$0.00 \pm 2.07$	$1.61 \pm 0.35$	0.01
10	$1.05 \pm 0.05$	$30.00 \pm 19.92$	$10.55 \pm 6.98$	0.04

The non-linear optimization process needs a qualified initial guess for the parameters since this optimization step is local. To obtain such initial guess, we discretize the differential equations by the trapezoidal rule (Aster et al. 2019). Fixing $V_{b0}$ and $C_s$ and the initial conditions temporarily, (see Table 1 for the values), thus *F* is linear with respect to the parameters and we simply solve a linear system of equations to obtain an initial guess for the subsequent non-linear parameter estimation procedure.

$$\begin{aligned} V_{b0}(z_{i+1}-z_i)&= V_{b0}\int _{t_i}^{t_{i+1}} z'(\tau ) d\tau \\&= \Delta t \left( C_s K_s - \left( K_s+K_b \right) \dfrac{z_{i+1}+z_i}{2}+K_b z_d \right) ,\\ V_{b0}(x_{i+1}-x_i)&= V_{b0}\int _{t_i}^{t_{i+1}} x'(\tau ) d\tau \\&= \Delta t\left( C_s K_s + \left( (K_s+K_b)\left( \dfrac{y_{i+1}+y_i}{2}- K_b \dfrac{x_{i+1}+x_i}{2} \right) \right) \right) ,\\ V_{d0}(y_{i+1}-y_i)&= V_{d0}\int _{t_i}^{t_{i+1}} y'(\tau ) d\tau \\&= \Delta t K_b\left( \dfrac{x_{i+1}+x_i}{2}-\dfrac{y_{i+1}+y_i}{2} \right) . \end{aligned}$$

Rearranging the terms, yields the linear system of equations,

49 $$\begin{aligned} \begin{bmatrix} C_s -\frac{1}{2}(z_{1}+z_0) &{} z_d-\frac{1}{2}(z_{1}+z_0) \\ C_s -\frac{1}{2}(z_{2}+z_1) &{} z_d-\frac{1}{2}(z_{2}+z_1) \\ \vdots &{} \vdots \\ C_s -\frac{1}{2}(x_{1}+x_0) &{} \frac{1}{2}(y_{1}+y_0-(x_{1}+x_0)) \\ C_s -\frac{1}{2}(x_{2}+x_1) &{} \frac{1}{2}(y_{2}+y_1-(x_{2}+x_1)) \\ \vdots &{} \vdots \\ 0 &{} \frac{1}{2}(x_{1}+x_0-(y_{1}+y_0))\\ 0 &{} \frac{1}{2}(x_{2}+x_1-(y_{2}+y_1))\\ \vdots &{} \vdots \\ \end{bmatrix} \begin{bmatrix} K_s \\ K_b \end{bmatrix} = \frac{1}{\Delta t} \begin{bmatrix} V_{b0}(z_{1}-z_{0}) \\ V_{b0}(z_{2}-z_{1}) \\ \vdots \\ V_{b0}(x_{1}-x_{0}) \\ V_{b0}(x_{2}-x_{1}) \\ \vdots \\ V_{d0}(y_{1}-y_{0}) \\ V_{d0}(y_{2}-y_{1}) \\ \vdots \end{bmatrix}. \end{aligned}$$

We compute the global, linear least square estimate for the three systems, SP, MP and the coupled system. We use the solution from the linear system in (49) as initial condition for $K_s$ and $K_b$, the initial conditions are initialized equal to the first measurement point, and $V_{b0}$ given by Table 1 as initial guess. Thereafter, we perturbed initial guesses, where we draw samples from the uniform distribution on the interval specified in Table 2 as initial guesses. The procedure showed that the optimization process is robust as the computed estimates in all simulations returned the same estimates.

**Table 4:** MP parameter values corresponding to Fig. 5 with 95% confidence interval for each parameter estimate specified

Patient	$C_s$ (mmol/L)	$K_s$ (L/h)	$K_b$ (L/h)	$V_{b0}$ (L)	RMSE
1	$2.19 \pm 0.15$	$2.61 \pm 0.65$	$6.89 \pm 1.19$	$10.05 \pm 2.83$	0.06
2	$1.32 \pm 0.04$	$5.01 \pm 0.61$	$8.39 \pm 0.62$	$12.31 \pm 1.71$	0.03
3	$1.36 \pm 0.12$	$8.74 \pm 3.74$	$7.62 \pm 1.44$	$12.09 \pm 7.15$	0.06
4	$1.48 \pm 0.13$	$6.72 \pm 2.78$	$8.59 \pm 1.62$	$22.32 \pm 10.08$	0.06
5	$1.62 \pm 0.03$	$4.65 \pm 0.33$	$10.17 \pm 0.42$	$18.40 \pm 1.35$	0.02
6	$1.60 \pm 0.15$	$4.22 \pm 1.55$	$7.28 \pm 1.67$	$10.57 \pm 4.42$	0.06
7	$1.75 \pm 0.15$	$4.10 \pm 2.48$	$7.04 \pm 1.22$	$31.07 \pm 16.65$	0.06
8	$1.28 \pm 0.19$	$2.41 \pm 1.63$	$3.89 \pm 1.39$	$10.17 \pm 7.14$	0.08
9	$1.07 \pm 0.10$	2$8.88 \pm 40.06$	$5.83 \pm 0.98$	$59.99 \pm 71.28$	0.06
10	$1.22 \pm 0.04$	$9.57 \pm 1.95$	$7.15 \pm 0.47$	$21.88 \pm 4.71$	0.03

**Table 5:** CP parameter values corresponding to Fig. 6 with 95% confidence interval for each parameter estimate specified

Patient	$C_s$ (mmol/L)	$K_s$ (L/h)	$K_b$ (L/h)	$V_{b0}$ (L)	RMSE
1	$2.20 \pm 0.16$	$2.72 \pm 0.15$	$6.85 \pm 0.63$	$9.09 \pm 1.00$	0.06
2	$1.32 \pm 0.06$	$5.30 \pm 0.05$	$8.26 \pm 0.75$	$10.98 \pm 0.63$	0.03
3	$1.38 \pm 0.14$	$8.00 \pm 0.11$	$8.05 \pm 2.66$	$10.70 \pm 1.32$	0.06
4	$1.92 \pm 0.18$	$3.58 \pm 0.17$	$7.88 \pm 1.09$	$22.68 \pm 1.90$	0.07
5	$1.69 \pm 0.16$	$4.84 \pm 0.16$	$8.68 \pm 1.63$	$16.76 \pm 1.60$	0.06
6	$2.70 \pm 0.26$	$1.43 \pm 0.23$	$6.22 \pm 0.42$	$11.78 \pm 2.05$	0.08
7	$1.64 \pm 0.18$	$4.43 \pm 0.18$	$6.62 \pm 7.14$	$49.46 \pm 1.57$	0.08
8	$1.32 \pm 0.21$	$2.28 \pm 0.19$	$4.26 \pm 1.30$	$8.06 \pm 1.37$	0.08
9	$1.23 \pm 0.10$	$10.42 \pm 0.09$	$5.90 \pm 5.22$	$55.18 \pm 0.88$	0.05
10	$1.21 \pm 0.07$	$10.65 \pm 0.06$	$7.06 \pm 2.88$	$20.01 \pm 0.67$	0.04

## Results and discussion

### Analytical results and discussion

The globally increasing number of patients with kidney failure implies a growing demand for dialysis to control toxin levels. Phosphate is naturally occurring in the diet and intake of phosphate binders is not sufficient to reduce phosphate for patients with lacking renal function leaving hemodialysis as a main method for phosphate reduction. Phosphate kinetics of SP and MP dialysis is investigated from a mathematical modelling perspective and analytical solutions are derived including rebound succeeding treatment. Main results obtained here are the explicit solution of the phosphate concentration during hemodialysis in typical clinical settings with or without ultrafiltration included [Eqs. (6) and (8)] showing exponential saturation. A transportable dialysis unit where the dialysate is reused is a useful alternative to traditional dialysis methods, coined MP dialysis. Main results provided here is the derivation of the governing differential equations for MP dialysis, Eq. (12), and the analytical solutions given by Eqs. (14) and (17). Neglecting the small contribution from ultrafiltration simpler formula are provided (42), (32) showing a rebound in the phosphate concentration in the plasma and extracellular fluid prior to the end of dialysis treatment.

For typical parameter values, the convective term is shown to be negligible in both MP and SP dialysis. This implies that the resulting phosphate concentrations follow mono - or biexponential behaviour which is explicitly expressed in terms of the original parameters. For SP and MP, a rebound effect is expected post treatment, again given by exponential saturation, Eq. (44).

These analytical solutions are important contributions to understanding phosphate kinetics during and between dialysis sessions. These equations can be used by clinicians to compare to clinical data and infer the patient specific parameters such as $K_s$. Phosphate concentration is found to exponentially decline in the SP model which implies that numerous short HD sessions is predicted to be more efficient than less frequent long HD interventions which is in line with clinical experience (Agar et al. 2011; Kooistra 2003). For MP the benefit of short term HD is amplified by the increased phosphate levels in the dialysate. However, each MP session involves additional time for setting up equipment and cleaning afterwards, which must be considered for planning optimal sessions.

The parameter $K_s$ is an important patient specific parameter. A higher value of $K_s$ implies a more efficient reduction of phosphate in the patient during dialysis. However, this also ensures a faster rebound after dialysis [Eq. (44)]. The post dialysis rebound equation may provide the simplest way to estimate $K_s$ which has not been clinically recognised before: The value $C_s$ can be estimated from a baseline measurement, $V_{b0}$ from bioimpedance, which leaves $K_s$ as the only unknown parameter in Eq. (44).

The SP model is used by Debowska et al. (2015) and one of their key findings is the correlation between $K_s$ and the relative change in phosphate before and after a HD session. This correlation can be understood in terms of the analytical solution formula [Eq. (8)] as follows: Assuming steady state before the HD session, $x(0)=C_s$, and neglecting the small contribution from the dialysate i.e. letting $C_{d0}=0$ we obtain

50 $$\begin{aligned} \frac{x(T)}{x(0)}=\frac{K_b}{K_s+K_b}\exp \left( -\frac{K_s+K_b}{V_{b0}}T\right) +\frac{K_s}{K_s+K_b}. \end{aligned}$$

At the end of treatment the exponential term may be approximated by the Taylor expansion to second order

51 $$\begin{aligned} \frac{x(T)}{x(0)}\approx \left( 1-\frac{K_b}{V_{b0}}T+\frac{1}{2}\left( \frac{K_bT}{V_{b0}} \right) ^2 \right) +\frac{1}{2}K_b\left( \frac{T}{V_{b0}} \right) ^2K_s , \end{aligned}$$

showing a positive, linear correlation between $\frac{x(T)}{x(0)}$ and $K_s$. Agar et al. (2011) define the rebound after a treatment of duration *T* by

52 $$\begin{aligned} {\text {rebound}}(t)=\frac{x(t+T)-x(T)}{x(T)}\cdot 100{\%} \end{aligned}$$

They compare short ($T=2$h) and conventional ($T=4$h) rebound, and find that they have very similar rebound curves. We can provide an explicit formula for this rebound

53 $$\begin{aligned} {\text {rebound}}(t)=\frac{C_s-x_T}{x_T}\left( 1-\exp \left( -\frac{K_s t}{V_{b0}} \right) \right) \cdot 100{\%}. \end{aligned}$$

From the rebound data in Agar et al. (2011), it is clear that for $T=2$h, the phosphate level has reached a plateau i.e. $x_T$ is constant, which is to be expected for sufficiently long time by Eq. (8). This means that $x_T$ may be considered constant for any $T\ge 2$ guaranteeing equal rebound curves for short and conventional HD, and the model predicts the same rebound for long HD i.e. 8 h. For $x_T$ being at the plateau level and neglecting the small contribution from $C_{d0}$, Eq. (53) implies

54 $$\begin{aligned} {\text {rebound}}(t)=\frac{K_b}{K_s}\left( 1-\exp \left( -\frac{K_s t}{V_{b0}} \right) \right) \cdot 100{\%}. \end{aligned}$$

which is valid for $T\ge 2$. This formula explains the rebound observed by Agar et al. (2011) and gives a direct parametric expression that may be used for parameter estimation provided rebound data is available. In particular, if $V_{b0}$ can be estimated from bioimpedance, then the rebound curve (54) calibrated to clinical data is sufficient to determine $K_b$ and $K_s$. Therefore, data of the rebound may provide a simple way to estimate $K_s$. Published studies often lack rebound data, or include only a single data point (Agar et al. 2015; Daugirdas 2018; Debowska et al. 2015) but we suggest to use rebound data in the future.

**Fig. 4:**
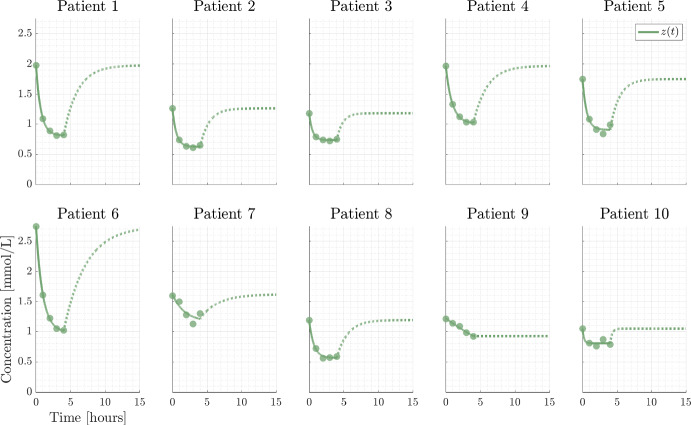
SP dialysis. Data points are represented by dots, the full and dashed lines are the SP model, Eq. (45), and post dialysis rebound. The parameter values are listed in Table 3

**Fig. 5:**
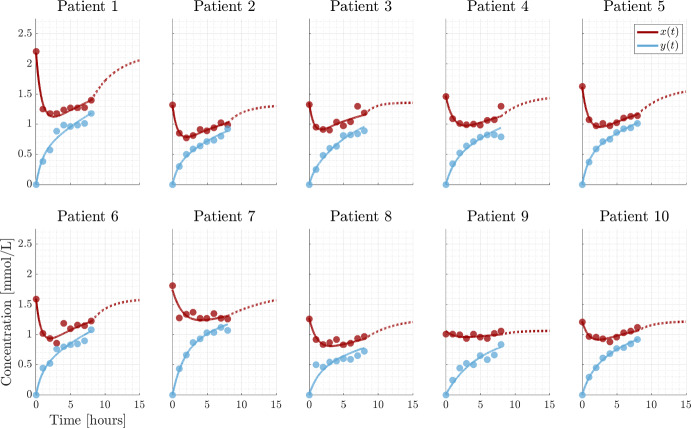
MP dialysis. Data points are represented by dots, the full and dashed lines are MP model (Eqs. 46–47), and post dialysis rebound. Parameter values are given in Table 4

**Fig. 6:**
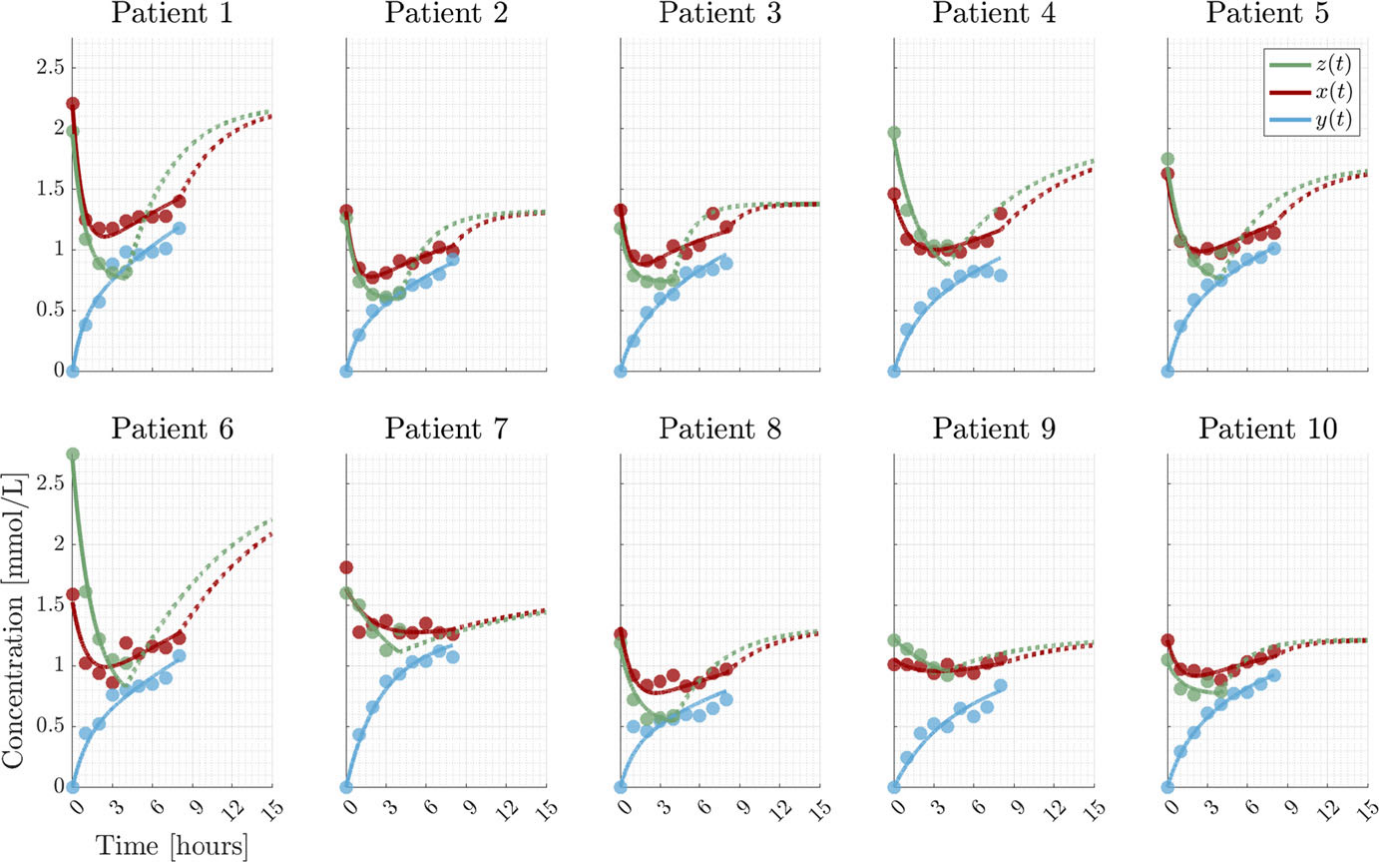
CP dialysis. Data points are represented by dots, the full and dashed lines are the SP and MP models, Eqs. (45–47), with identical parameter values and stipulated curves are post dialysis rebound. Parameter values are given by Table 5

### Computational results and discussion

The same cohort of ten patients have been exposed to SP and MP treatment allowing for use of both data sets to estimate the physiological parameters (the CP model). The successful calibration of CP to clinical data implies consistency between the SP and MP models. The findings validate the underlying model assumptions and the resulting analytical solutions, Eqs. (8) and (42).

The model trajectories for SP, MP and CP calibrated to clinical data are shown in Figs. 4, 5 and 6 with corresponding parameter values in Tables 3, 4 and 5, providing good agreement between models and data. Each estimated parameter values is attributed a 95% confidence interval calculated from the square root of the diagonal elements of the covariance matrix for the linearized problem. We estimate $C_s$ based on the assumption that the patient is in steady state at time $t =0$. However, the initial measurements can be encumbered with noise and may not be at steady state (see the large difference between $z_0$ and $x_0$ for patient 6). To relax the assumption, one could measure the relapse to achieve more information about $C_s$. Thus, $C_s$ is considered a parameter not only relying on the initial measurement. Furthermore, the uncertain steady state assumption at time 0 is avoided. Another possibility is to use a Bayesian approach where prior information about e.g. $C_s$ is included to improve the identifiability of the parameters (Bangsgaard et al. 2023; Vanlier et al. 2013).

The models being structurally identifiable (with $V_{b0}$ fixed at the value obtained by the clinical measurement in case of SP) may not be practically identifiable. From Table 3 we generally see small uncertainties on the estimated parameter values for SP except for patient 7 and to minor degree for patient 10. Patient 7 may be considered an outlier, since the first four data point lies approximately on a straight line. Thus, the exponential fit does not capture this well, which is also reflected by the value of the RMSE in Table 3.

For MP, the uncertainties are generally slightly larger without being alarming. However, the uncertainty for patient 7 is slightly better than for SP while the uncertainties for patient 9 has worsened, which is likely due to the additional data points. For MP, the data for patient 9 approximately lie on a horizontal line indicating problems with this specific MP dialysis treatment for unknown reasons.

The troublesome uncertainties in the case of SP and MP are resolved for CP where all uncertainties are acceptable. These observations are supported by the relatively small RMSE for all patients.

The general trend of slightly elevated uncertainties for MP compared to SP may be explained by the increase in the number of estimated parameters and the inclusion of additional data. The larger uncertainties for patient 9 are likely due to the upper bound for the blood volume $V_{b0}$ being reached.

The uncertainties and corresponding estimates for patient 7 during SP and patient 9 for MP are on the limit of being problematic while none are for CP. Thus, the parameters are generally practically identifiable from the available data at least when excluding the two limiting cases.

Ideally, we should obtain the same value for the estimated parameter $K_s$ in both SP and MP as it represents the same patient specific transfer rate. However, this is not the case primarily because the parameter $V_{b0}$ for SP is non-identifiable. This is a motivation for introducing CP, where both SP and MP are considered simultaneously having the same physiological parameters. Here we obtain a more accurate estimate for the parameters e.g., $K_s$.

Optimal control of dialysis treatment would be an interesting next step to address for applied mathematicians interested in assisting nephrologist to help improve the situation for the millions of people with chronic kidney failure.

## Data Availability

No new data was generated, we relied on published data (Heaf et al. 2013).
